# Two new species of *Eretmocerus* Haldeman (Hymenoptera: Aphelinidae) parasitizing *Aleurolobus rhododendri* Takahashi and *Dialeuropora decempunctata* (Quaintance & Baker) (Hemiptera: Aleyrodidae) from Taiwan

**DOI:** 10.3897/BDJ.4.e7713

**Published:** 2016-06-07

**Authors:** Samantha E. Ward, Yuan Tung Shih, Chiun-Cheng Ko, Andrew Polaszek

**Affiliations:** ‡ Natural History Museum, London, United Kingdom; § National Taiwan University, Taipei, Taiwan

**Keywords:** taxonomy, whitefly parasitoid, *Bemisia tabaci*-complex

## Abstract

**Background:**

Species of *Eretmocerus* Haldeman develop as primary ecto-endoparasites of whiteflies (Rose and Rosen 1992). Currently, the genus *Eretmocerus* comprises 86 species worldwide, of which 11 species have been previously recorded from Taiwan (Shih et al. 2015). Despite having been recently revised for Taiwan, two new species are here added to the Taiwan fauna.

**New information:**

Two new species, *Eretmocerus garrywardi* Ward **sp. nov.** and *Eretmocerus liangyihchoui* Shih **sp. nov.** found parasitizing *Aleurolobus rhododendri* Takahashi and *Dialeuropora decempunctata* respectively, are described. A key to females of *Eretmocerus* species occurring in Taiwan is provided.

## Introduction

Species of *Eretmocerus* Haldeman develop as primary ecto-endoparasites of whiteflies (Rose and Rosen 1992). Currently, the genus *Eretmocerus* comprises 86 species worldwide Noyes 2015, of which 11 species have been previously recorded from Taiwan (Shih et al. 2015). The current paper brings the total number of Taiwanese *Eretmocerus* species to 13.

## Materials and methods

### Survey, collection and identification of parasitoid hosts

A series of surveys were undertaken from 2004 to 2014 for collection of parasitoid host whiteflies, scale insects and aphids in Taiwan (see Shih et al. 2015). Whiteflies were identified by K.C. Chou (Taiwan Agricultural Research Institute) & C.C. Ko from National Taiwan University, Taiwan.

### Terminology

Morphological terminology and the format for species descriptions follow Shih et al. 2015. Basic morphological characters for females are: Antenna: length of clava (C), especially relative to its width; length and shape of first funicle segment (F1); length and shape of second funicle segment (F2); length of pedicel (P); length of radicle (R); length of scape (S). Wing: length of fore wing (L), length of marginal vein (M), length of submarginal vein (SM), length of stigmal vein (ST), greatest width of disc (W), especially relative to the longest posterior alar fringe. Mesosoma: length of mid lobe (ML); greatest width of mid lobe (WM); length of scutellum (SC); greatest width of scutellum (WS). Gaster (metasoma minus petiole): length of gaster (G), arrangement of paired setae on tergites 2-6. Leg: length of mid tibia (MT).

Line drawings were made using an Olympus BX51 compound microscope located in the Dept. of Entomology, National Taiwan University (NTU), Taiwan.

### Depositories

The holotypes and paratypes of the new species are deposited in the Department of Entomology, National Taiwan University, and Taiwan Agriculural Research Institute, Taiwan. Paratypes of both species are also deposited in the Natural History Museum, London, UK.

### Abbreviations

KCC: K.C. Chou (collector)

BMNH: Natural History Museum, London UK

NTU: National Taiwan University

TARI: Taiwan Agricultural Research Institute

YTS: Y.T. Shih (collector)

## Taxon treatments

### Eretmocerus garrywardi

Ward 2016 sp. n.

urn:lsid:zoobank.org:act:370E8E1E-71A9-4BD4-9CA7-F9DA814D188F

#### Materials

- **Type status:** Holotype. **Occurrence:** recordedBy: Yuan Tung Shih; individualID: 5981/298; individualCount: 1; sex: ♀; lifeStage: adult; previousIdentifications: *Dialeuropora decempunctata* on *Machilus zuihensis* 5981/298; **Taxon:** scientificName: Eretmocerus garrywardi; **Location:** country: Taiwan; county: Pingtung; locality: Wanluan; **Event:** eventDate: 29-XI-2005; **Record Level:** institutionCode: Natural History Museum, London, UK (BMNH)
- **Type status:** Paratype. **Occurrence:** occurrenceRemarks: ex *Bemisia tabaci* on *Emilia sonchifolia*; recordedBy: Yuan Tung Shih; individualCount: 5; sex: 4 ♀ 1 ♂; lifeStage: adult; **Taxon:** scientificName: Eretmocerus garrywardi; **Location:** country: Taiwan; county: Taoyuan; locality: Guanyin; **Event:** eventDate: 12-IX-2010; **Record Level:** institutionCode: National Taiwan University, Taipei, Taiwan (NTU)
- **Type status:** Paratype. **Occurrence:** occurrenceRemarks: ex *Dialeuropora decempunctata* on *Machilus zuihensis*; recordedBy: Yuan Tung Shih; individualID: 5981/298; individualCount: 1; sex: ♀; lifeStage: adult; **Taxon:** scientificName: Eretmocerus garrywardi; **Location:** country: Taiwan; county: Pingtung; locality: Wanluan; **Event:** eventDate: 29-XI-2005; **Record Level:** institutionCode: Natural History Museum, London, UK (BMNH)
- **Type status:** Paratype. **Occurrence:** occurrenceRemarks: ex *Dialeuropora* sp. on *M. zuihensis*; recordedBy: Yuan Tung Shih; individualCount: 1; sex: ♀; lifeStage: adult; **Taxon:** scientificName: Eretmocerus garrywardi; **Location:** country: Taiwan; municipality: Taipei City; locality: Beitou; **Event:** eventDate: 18-XII-2008; **Record Level:** institutionCode: National Taiwan University, Taipei, Taiwan (NTU)
- **Type status:** Paratype. **Occurrence:** occurrenceRemarks: ex *D. decempunctata* on *M. zuihensis*; recordedBy: Yuan Tung Shih; individualCount: 3; sex: ♀; lifeStage: adult; **Taxon:** scientificName: Eretmocerus garrywardi; **Location:** country: Taiwan; county: Changhua; locality: Baguashan; **Event:** eventDate: 17-I-2011; **Record Level:** institutionCode: National Taiwan University, Taipei, Taiwan (NTU)
- **Type status:** Paratype. **Occurrence:** occurrenceRemarks: ex *D. decempunctata* on *M. zuihensis*; recordedBy: Yuan Tung Shih; individualCount: 1; sex: ♀; lifeStage: adult; **Taxon:** scientificName: Eretmocerus garrywardi; **Location:** country: Taiwan; municipality: Taichung; locality: Dakeng; **Event:** eventDate: 3-VI-2013; **Record Level:** institutionCode: National Taiwan University, Taipei, Taiwan (NTU)
- **Type status:** Paratype. **Occurrence:** occurrenceRemarks: ex *D. decempunctata* on *M. zuihensis*; recordedBy: Yuan Tung Shih; individualCount: 2; sex: ♀; lifeStage: adult; **Taxon:** scientificName: Eretmocerus garrywardi; **Location:** country: Taiwan; municipality: New Taipei City; locality: Linkou; **Event:** eventDate: 10-VI-2013; **Record Level:** institutionCode: National Taiwan University, Taipei, Taiwan (NTU)
- **Type status:** Paratype. **Occurrence:** occurrenceRemarks: ex *D. decempunctata* on *Rubus* sp.; recordedBy: Ko Chiun-Cheng; individualCount: 1; sex: ♀; lifeStage: adult; **Taxon:** scientificName: Eretmocerus garrywardi; **Location:** country: Taiwan; county: Nantou; locality: Puli; **Event:** eventDate: 8-XII-1995; **Record Level:** institutionCode: Taiwan Agricultural Research Institute, Wufeng, Taiwan (TARI)

#### Description

**Female holotype.**

Body length: 0.90 mm.

**Colour.** Head and body entirely yellow, wings hyaline. Legs pale yellow except basal margins of mid and hind femora darker.

**Morphology.**

**Figure 1.**
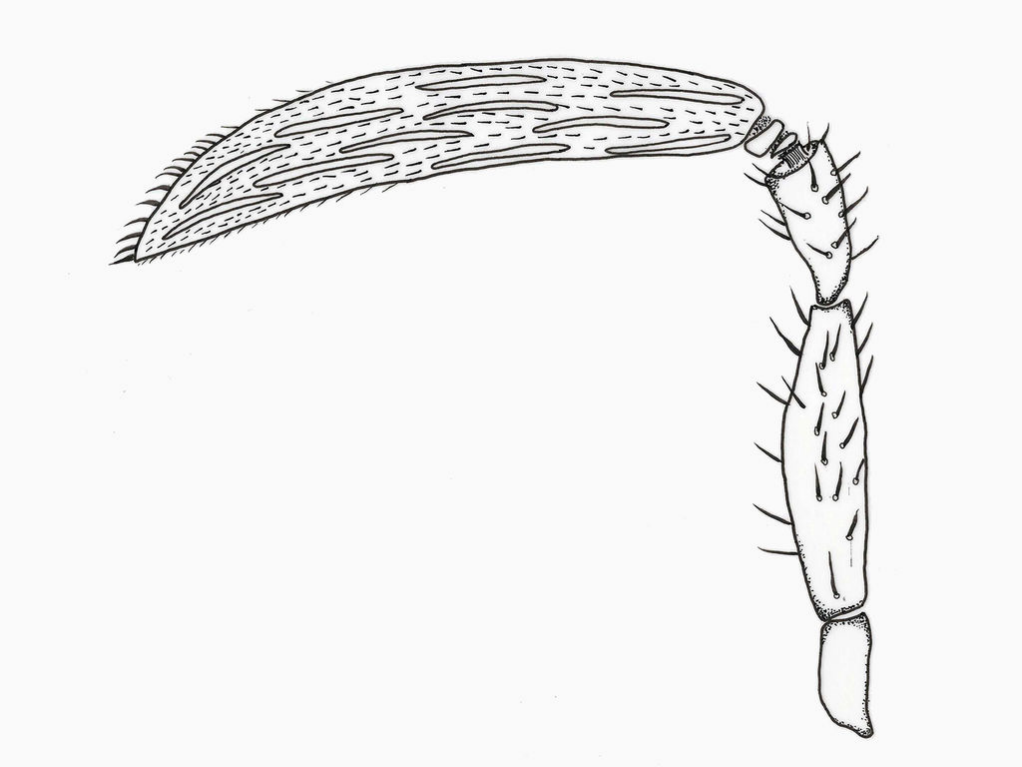
*Eretmocerus garrywardi* female antenna

**Antenna** (Fig. 1). Radicle 2.5× as long as wide; scape 4.8× as long as wide, 3.8× as long as radicle, 3.2× pedicel length, 0.7× clava length; pedicel 1.8× as long as wide, 1.2× as long as radicle, 0.3× scape length; funicle I and 2 anelliform / transverse; clava pointed, blade-shaped, 4.6× as long as the greatest width 1.5× scape length.

**Figure 2.**
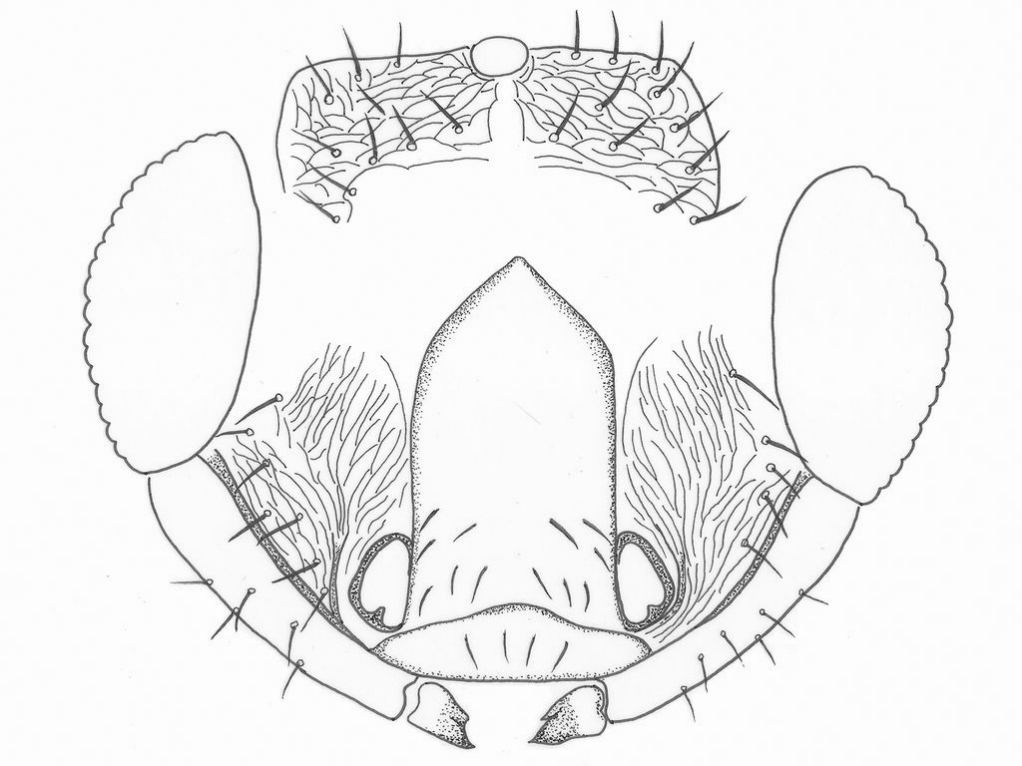
*Eretmocerus garrywardi* head, front view

**Figure 3.**
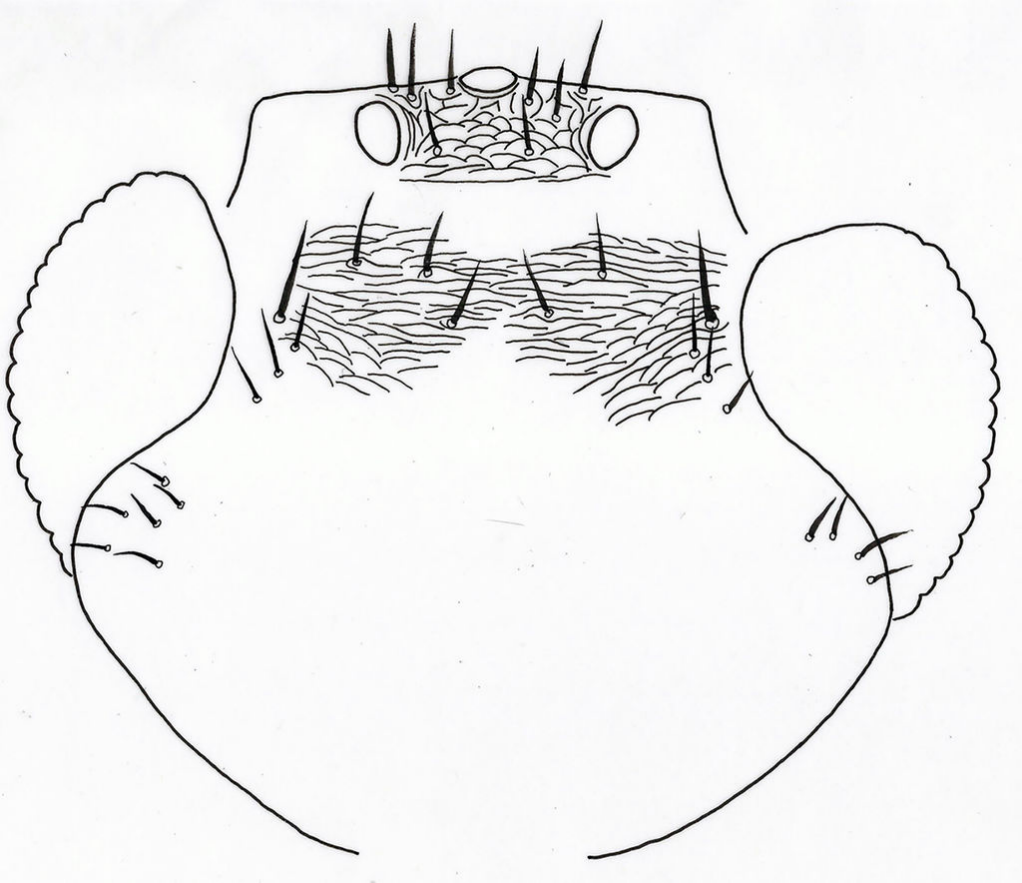
*Eretmocerus garrywardi* head, posterior view

**Head** (Figs 2, 3). Vertex with 11–13 pairs of setae; face and occiput with transverse substrigose sculpture, interscrobal area vertically strigose; face with 12–16 pairs of setae; supraclypeal area with 10–18 setae; clypeus with 2+2 setae. Mandible unusually small.

**Figure 4.**
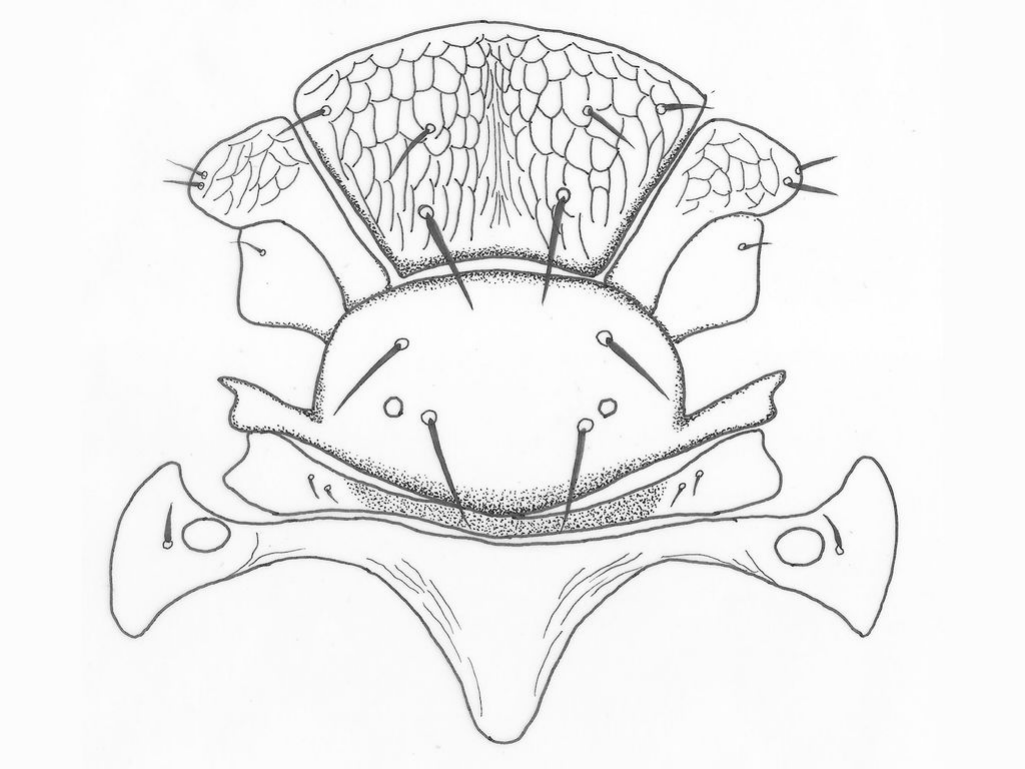
*Eretmocerus garrywardi* dorsal mesosoma excluding pronotum

**Mesosoma** (Fig. 4). Mid lobe of mesoscutum with 6 setae, anterior part with cellular reticulate sculpture, remainder with faint elongate reticulations; side lobe with 2 setae, anterior margins with faint reticulations; axilla with 1 seta, faintly reticulate; scutellum with 4 setae, anterior pair shorter, 0.8x posterior pair length, 2 placoid sensilla placed laterally equidistant from both paired setae; frenal arms long, exceeding metanotum; metanotum narrower centrally than propodeum; propodeum elongate, with faint transverse reticulations, central lobe broad and smooth.

**Wings** (Fig. 6). Fore wing 2.4× as long as maximum width of disc; longest posterior alar fringe 0.3× maximum disc width; marginal vein with 2-3 long setae; wing disc with setae clearly arranged in lines, with linea calva present.

**Legs**. Fore coxa with 3 setae on lateral margin; trochanter with 2 short setae and without reticulations. Mid coxa with 4 setae; mid tibial spur 0.5× basitarsus length. Hind coxa with 2 pairs of setae, 1 short pair placed close to the base; trochanter with 3 setae; hind tibial spur 0.5× basitarsus length.

**Figure 5.**
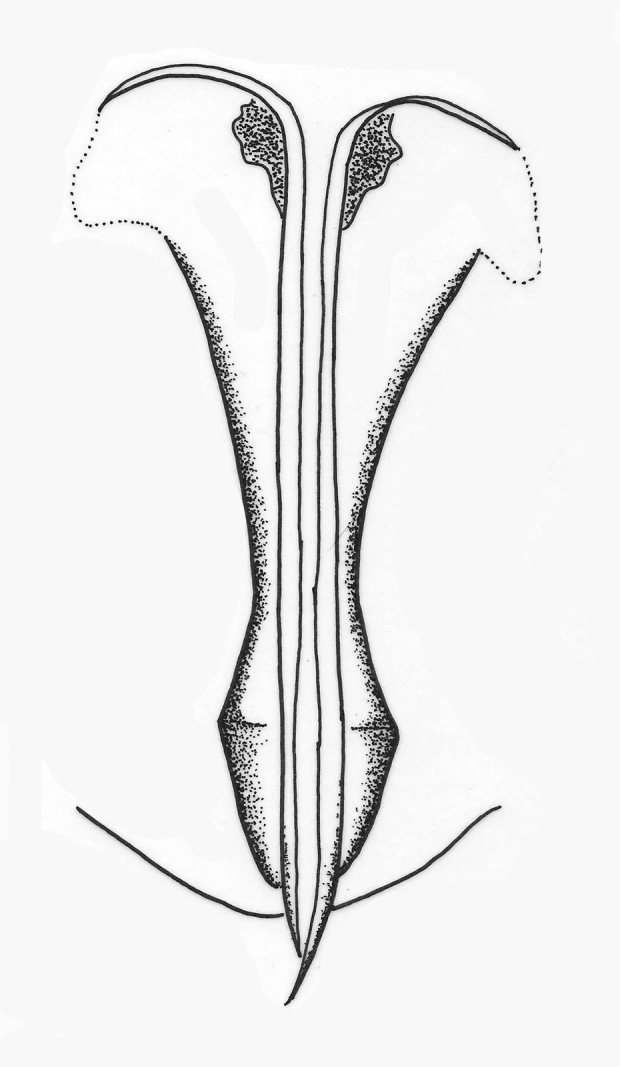
*Eretmocerus garrywardi* ovipositor

**Figure 6.**
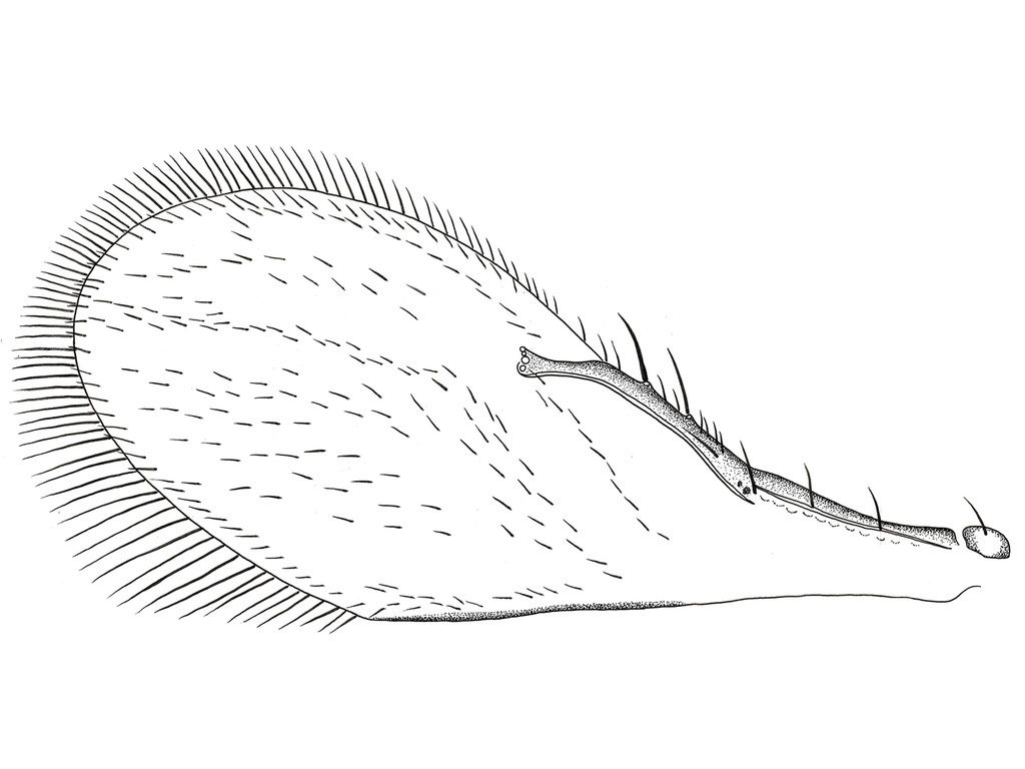
*Eretmocerus garrywardi* fore wing

**Gaster**. Gastral tergite **1** with reticulations on lateral margins; tergites 1–6 with paired setae as follows: 1, 1, 1, 2, 3, 3-4; syntergum (T7) with 4 setae; ovipositor (Fig. 5) weakly exserted, nearly equal to clava length, 1.7× scape length, 1.1× mid tibia length.

**Male.**

Colour. Similar to female, except middle area of mid lobe of mesoscutum and scutellum pale brown. Marginal vein and Submarginal vein pale brown.

Head as female, except interscrobal area circular, fewer setae on vertex, face and occiput. Antenna with 3 segments, clava cylindrical, acutely pointed at apex. Mesosoma as female, except mid lobe of mesoscutum, and scutellum, with strong reticulate sculpture. Genitalia with elongate aedeagus, phallobase present.

#### Diagnosis

*Eretmocerus garrywardi* **sp.n.** can be distinguished from other species in the genus by the following combination of characters: Antennal clava laminate and blade-shaped. F1 ring-like; F2 triangular-trapezoid. Mid lobe of mesoscutum with 6 setae; side lobe of mesoscutum with 2 setae; propodeum elongate. Fore wing with an unusual linear arrangement of setae; marginal vein longer than stigmal vein; marginal fringe more than 0.25x wing width.

#### Etymology

Named for Garry Ward, father of the first author.

#### Distribution

**TAIWAN**: New Taipei City: Beitou; Taoyuan: Guanyin, Linkou; Taichung City: Dakeng, Puli; Changhua County: Baguashan; Pingtung County: Wanluan.

#### Biology

**Hosts**: Hemiptera: Aleyrodidae: *Dialeuropora decempunctata* (Quaintance & Baker), *Bemisia tabaci* (Gennadius).

### Eretmocerus liangyihchoui

Shih, Ward & Polaszek, 2016 sp. n.

urn:lsid:zoobank.org:act:C2BBC5D6-BB85-4880-BC2D-579607A84B8E

#### Materials

- **Type status:** Holotype. **Occurrence:** occurrenceRemarks: ex *Aleurolobus rhododendri* on *Bauhinia variegata*; recordedBy: Ko Chiun-Cheng; individualCount: 1; sex: female; lifeStage: adult; **Taxon:** scientificName: Eretmocerus liangyihchoui; **Location:** country: Taiwan; locality: Wufeng District, Taichung City, Taiwan Agricultural Research Institute; **Event:** eventDate: 27-X-1993; **Record Level:** institutionCode: Taiwan Agricultural Research Institute, Wufeng, Taiwan (TARI)
- **Type status:** Paratype. **Occurrence:** occurrenceRemarks: ex *Aleurolobus rhododendri* on *Bauhinia variegata*; recordedBy: Ko Chiun-Cheng; individualCount: 1; sex: female; lifeStage: adult; **Taxon:** scientificName: Eretmocerus liangyihchoui; **Location:** country: Taiwan; locality: Nantou; **Event:** eventDate: 28-X-1993; **Record Level:** institutionCode: Taiwan Agricultural Research Institute, Wufeng, Taiwan (TARI)
- **Type status:** Paratype. **Occurrence:** occurrenceRemarks: ex *A. rhododendri* on *Averrhoa carambola*; recordedBy: Ko Chiun-Cheng; individualCount: 1; sex: female; lifeStage: adult; **Taxon:** scientificName: Eretmocerus liangyihchoui; **Location:** country: Taiwan; locality: Chiai; **Event:** eventDate: 5-XI-1993; **Record Level:** institutionCode: Taiwan Agricultural Research Institute, Wufeng, Taiwan (TARI)
- **Type status:** Paratype. **Occurrence:** occurrenceRemarks: ex *A. rhododendri* on *A. carambola*; recordedBy: Ko Chiun-Cheng; individualCount: 3; sex: female; lifeStage: adult; **Taxon:** scientificName: Eretmocerus liangyihchoui; **Location:** country: Taiwan; locality: Wufeng; **Event:** eventDate: 16-XI-1993; **Record Level:** institutionCode: Taiwan Agricultural Research Institute, Wufeng, Taiwan (TARI)
- **Type status:** Paratype. **Occurrence:** occurrenceRemarks: ex *A. rhododendri* on *A. carambola*; recordedBy: Ko Chiun-Cheng; individualCount: 1; sex: female; lifeStage: adult; **Taxon:** scientificName: Eretmocerus liangyihchoui; **Location:** country: Taiwan; locality: Wufeng; **Event:** eventDate: 16-XI-1993; **Record Level:** institutionCode: Natural History Museum, London, UK (BMNH)
- **Type status:** Paratype. **Occurrence:** occurrenceRemarks: ex *A. rhododendri* on *A. carambola*; recordedBy: Ko Chiun-Cheng; individualCount: 2; sex: female; lifeStage: adult; **Taxon:** scientificName: Eretmocerus liangyihchoui; **Location:** country: Taiwan; locality: Fengshan; **Event:** eventDate: 22-XI-1993; **Record Level:** institutionCode: Taiwan Agricultural Research Institute, Wufeng, Taiwan (TARI)
- **Type status:** Paratype. **Occurrence:** occurrenceRemarks: ex *A. rhododendri* on *Rhododendron formosanum*; recordedBy: Ko Chiun-Cheng; individualCount: 2; sex: female; lifeStage: adult; **Taxon:** scientificName: Eretmocerus liangyihchoui; **Location:** country: Taiwan; locality: Chushan; **Event:** eventDate: 29-XI-1993; **Record Level:** institutionCode: Taiwan Agricultural Research Institute, Wufeng, Taiwan (TARI)
- **Type status:** Paratype. **Occurrence:** occurrenceRemarks: ex *A. rhododendri* on *B. variegata*; recordedBy: Ko Chiun-Cheng; individualCount: 1; sex: female; lifeStage: adult; **Taxon:** scientificName: Eretmocerus liangyihchoui; **Location:** country: Taiwan; locality: Wufeng District, Taichung City, Taiwan Agricultural Research Institute; **Event:** eventDate: 8-XII-1993; **Record Level:** institutionCode: Taiwan Agricultural Research Institute, Wufeng, Taiwan (TARI)
- **Type status:** Paratype. **Occurrence:** occurrenceRemarks: ex *A. rhododendri* on *B. variegata*; recordedBy: Ko Chiun-Cheng; individualCount: 2; sex: female; lifeStage: adult; **Taxon:** scientificName: Eretmocerus liangyihchoui; **Location:** country: Taiwan; locality: Nantou; **Event:** eventDate: 23-XII-1993; **Record Level:** institutionCode: Taiwan Agricultural Research Institute, Wufeng, Taiwan (TARI)
- **Type status:** Paratype. **Occurrence:** occurrenceRemarks: ex *A. rhododendri* on *B. variegata* and *A. carambola*; recordedBy: Ko Chiun-Cheng; individualCount: 2; sex: female; lifeStage: adult; **Taxon:** scientificName: Eretmocerus liangyihchoui; **Location:** country: Taiwan; locality: Chunghsinghsintsun; **Event:** eventDate: 23-XII-1993; **Record Level:** institutionCode: Taiwan Agricultural Research Institute, Wufeng, Taiwan (TARI)
- **Type status:** Paratype. **Occurrence:** occurrenceRemarks: ex *A. rhododendri* on *B. variegata*; recordedBy: Ko Chiun-Cheng; individualCount: 3; sex: female; lifeStage: adult; **Taxon:** scientificName: Eretmocerus liangyihchoui; **Location:** country: Taiwan; locality: Wufeng District, Taichung City, Taiwan Agricultural Research Institute; **Event:** eventDate: 31-XII-1993; **Record Level:** institutionCode: 2♀ Natural History Museum, London, UK (BMNH), 1♀ Taiwan Agricultural Research Institute, Wufeng, Taiwan (TARI)
- **Type status:** Paratype. **Occurrence:** occurrenceRemarks: ex *A. rhododendri* on *A. carambola*; recordedBy: Ko Chiun-Cheng; individualCount: 2; sex: female; lifeStage: adult; **Taxon:** scientificName: Eretmocerus liangyihchoui; **Location:** country: Taiwan; locality: Wufeng; **Event:** eventDate: 6-I-1994; **Record Level:** institutionCode: Taiwan Agricultural Research Institute, Wufeng, Taiwan (TARI)
- **Type status:** Paratype. **Occurrence:** occurrenceRemarks: ex *A. rhododendri* on *B. variegata*; recordedBy: Ko Chiun-Cheng; individualCount: 1; sex: female; lifeStage: adult; **Taxon:** scientificName: Eretmocerus liangyihchoui; **Location:** country: Taiwan; locality: Wufeng District, Taichung City, Taiwan Agricultural Research Institute; **Event:** eventDate: 17-I-1994; **Record Level:** institutionCode: Taiwan Agricultural Research Institute, Wufeng, Taiwan (TARI)
- **Type status:** Paratype. **Occurrence:** occurrenceRemarks: ex *A. rhododendri* on *R. formosanum*; recordedBy: Ko Chiun-Cheng; individualCount: 3; sex: female; lifeStage: adult; **Taxon:** scientificName: Eretmocerus liangyihchoui; **Location:** country: Taiwan; locality: Yangmingshan; **Event:** eventDate: 27-I-1994; **Record Level:** institutionCode: Taiwan Agricultural Research Institute, Wufeng, Taiwan (TARI)
- **Type status:** Paratype. **Occurrence:** occurrenceRemarks: ex *A. rhododendri* on *B. variegata*; recordedBy: Ko Chiun-Cheng; individualCount: 1; sex: female; lifeStage: adult; **Taxon:** scientificName: Eretmocerus liangyihchoui; **Location:** country: Taiwan; locality: Wufeng District, Taichung City, Taiwan Agricultural Research Institute; **Event:** eventDate: 3-II-1994; **Record Level:** institutionCode: Taiwan Agricultural Research Institute, Wufeng, Taiwan (TARI)
- **Type status:** Paratype. **Occurrence:** occurrenceRemarks: ex *A. rhododendri* on *Pueraria lobata*; recordedBy: Ko Chiun-Cheng; individualCount: 1; sex: female; lifeStage: adult; **Taxon:** scientificName: Eretmocerus liangyihchoui; **Location:** country: Taiwan; locality: Wufeng; **Event:** eventDate: 10-III-1994; **Record Level:** institutionCode: Taiwan Agricultural Research Institute, Wufeng, Taiwan (TARI)
- **Type status:** Paratype. **Occurrence:** occurrenceRemarks: ex *Aleurocybotus* sp. on *Miscanthus floridulus*; recordedBy: Ko Chiun-Cheng; individualCount: 1; sex: female; lifeStage: adult; **Taxon:** scientificName: Eretmocerus liangyihchoui; **Location:** country: Taiwan; locality: Kungkuan; **Event:** eventDate: 20-III-1994; **Record Level:** institutionCode: Taiwan Agricultural Research Institute, Wufeng, Taiwan (TARI)
- **Type status:** Paratype. **Occurrence:** occurrenceRemarks: ex *A. rhododendri* on *A. carambola*; recordedBy: Ko Chiun-Cheng; individualCount: 1; sex: female; lifeStage: adult; **Taxon:** scientificName: Eretmocerus liangyihchoui; **Location:** country: Taiwan; locality: Mucha; **Event:** eventDate: 7-I-1995; **Record Level:** institutionCode: Taiwan Agricultural Research Institute, Wufeng, Taiwan (TARI)
- **Type status:** Paratype. **Occurrence:** occurrenceRemarks: ex *A. rhododendri* on *Rhododendron oldhamii*; recordedBy: Yuan Tung Shih; individualCount: 1; sex: female; lifeStage: adult; **Taxon:** scientificName: Eretmocerus liangyihchoui; **Location:** country: Taiwan; locality: Huwei; **Event:** eventDate: 14-IX-2005; **Record Level:** institutionCode: National Taiwan University, Taipei, Taiwan (NTU)
- **Type status:** Paratype. **Occurrence:** occurrenceRemarks: ex *A. rhododendri* on *R. oldhamii*; recordedBy: Yuan Tung Shih; individualCount: 1; sex: female; lifeStage: adult; **Taxon:** scientificName: Eretmocerus liangyihchoui; **Location:** country: Taiwan; locality: Linkou; **Event:** eventDate: 4-XI-2005; **Record Level:** institutionCode: National Taiwan University, Taipei, Taiwan (NTU)
- **Type status:** Paratype. **Occurrence:** occurrenceRemarks: ex *A. rhododendri* on *R. oldhamii*; recordedBy: Yuan Tung Shih; individualCount: 1; sex: female; lifeStage: adult; **Taxon:** scientificName: Eretmocerus liangyihchoui; **Location:** country: Taiwan; locality: Nantou; **Event:** eventDate: 18-I-2006; **Record Level:** institutionCode: National Taiwan University, Taipei, Taiwan (NTU)
- **Type status:** Paratype. **Occurrence:** occurrenceRemarks: ex *A. rhododendri* on *R. formosanum*; recordedBy: Yuan Tung Shih; individualCount: 1; sex: female; lifeStage: adult; **Taxon:** scientificName: Eretmocerus liangyihchoui; **Location:** country: Taiwan; locality: Ankeng; **Event:** eventDate: 28-XII-2008; **Record Level:** institutionCode: National Taiwan University, Taipei, Taiwan (NTU)
- **Type status:** Paratype. **Occurrence:** occurrenceRemarks: ex *A. rhododendri* on *R. formosanum*; recordedBy: Yuan Tung Shih; individualCount: 1; sex: female; lifeStage: adult; **Taxon:** scientificName: Eretmocerus liangyihchoui; **Location:** country: Taiwan; locality: Yangmingshan; **Event:** eventDate: 16-I-2009; **Record Level:** institutionCode: National Taiwan University, Taipei, Taiwan (NTU)

#### Description

**Female holotype.**

Body length: 1.1 mm.

**Colour.** Head yellow to pale brown. Mesosoma yellow, some individuals with mid lobe of mesoscutum and metanotum brown; propodeum dark brown, but paler laterally. Gaster yellow. Antenna yellow to pale brown. Wings hyaline, except marginal and submarginal vein brown. Legs pale yellow.

**Morphology.**

**Figure 7.**
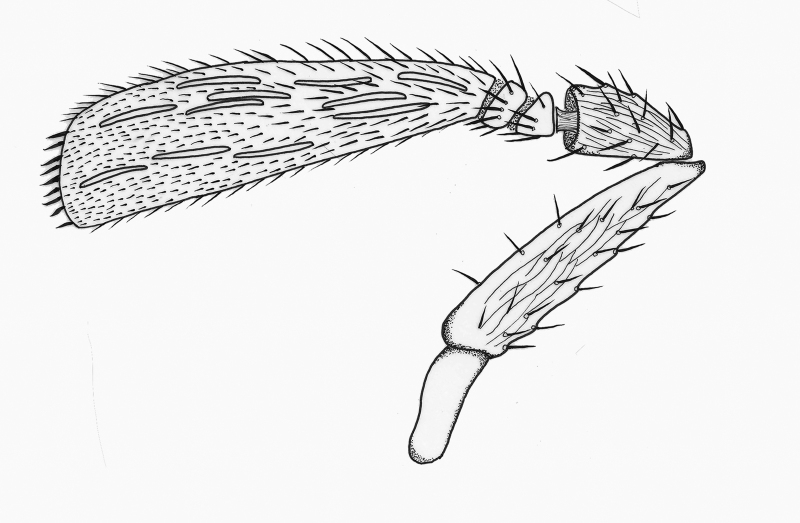
*Eretmocerus liangyihchoui* female antenna

**Antenna** (Fig. 7). Scape 4.5× as long as wide, 2.8× as long as radicle, 2.3× pedicel length, 0.7× clava length; pedicel 2.1× as long as wide, 1.2× as long as radicle, 0.4× scape length; funicle I trapezoid, dorsal length 0.3× ventral length; funicle II transverse rectangular, 2.4x as wide as long; clava clavate, much narrower at base than apex, 3.8-3.9× as long as greatest width, 1.5× scape length, 3.5× pedicel length; clava with 16-20 longitudinal sensilla.

**Figure 8.**
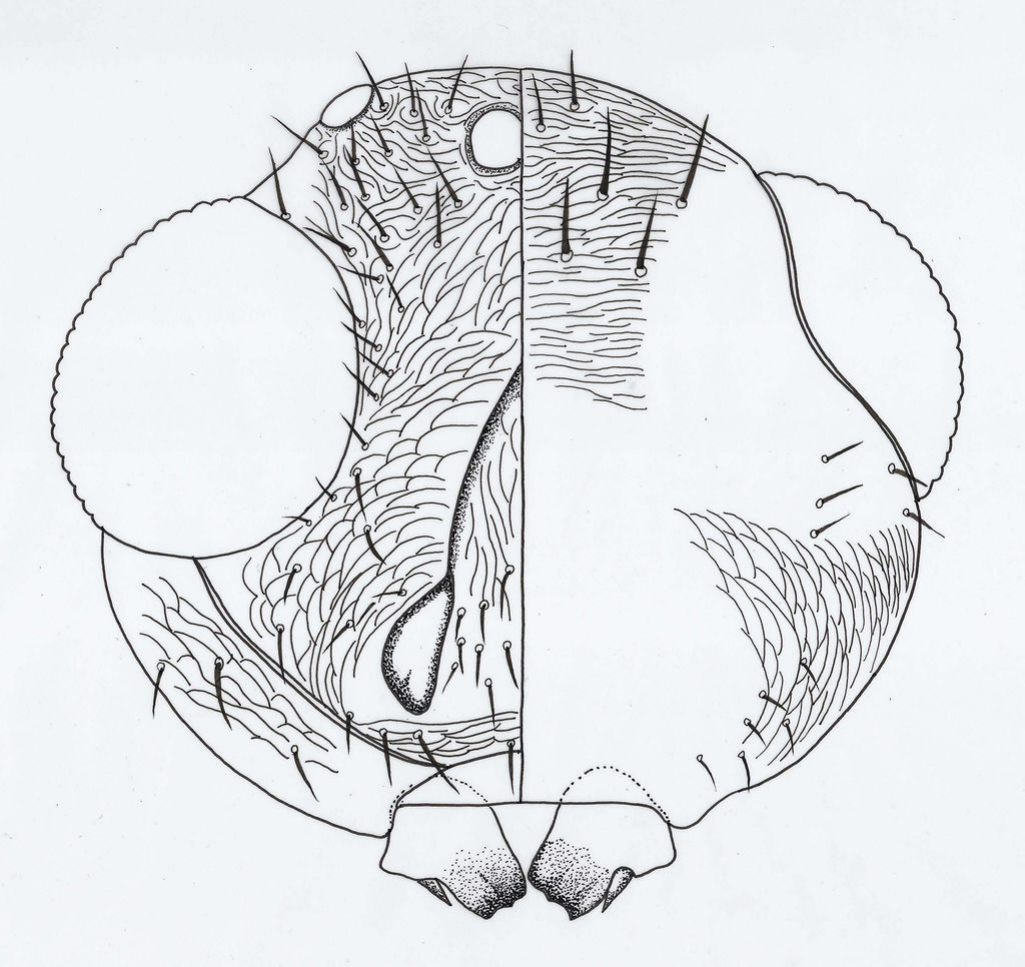
*Eretmocerus liangyihchoui* head, right half front view, left half posterior view

**Head** (Fig. 8). Vertex with 17–18 pairs of setae; face and occiput with transverse substrigose sculpture, interscrobal area vertically strigose; face with 10-12 pairs of setae; supraclypeal area with 13-15 setae; clypeus with 2+2 setae; upper posterior head with 14–16 setae, 3 pairs of long and robust setae present in a row across the head; lower posterior head with 12+12 setae.

**Figure 9.**
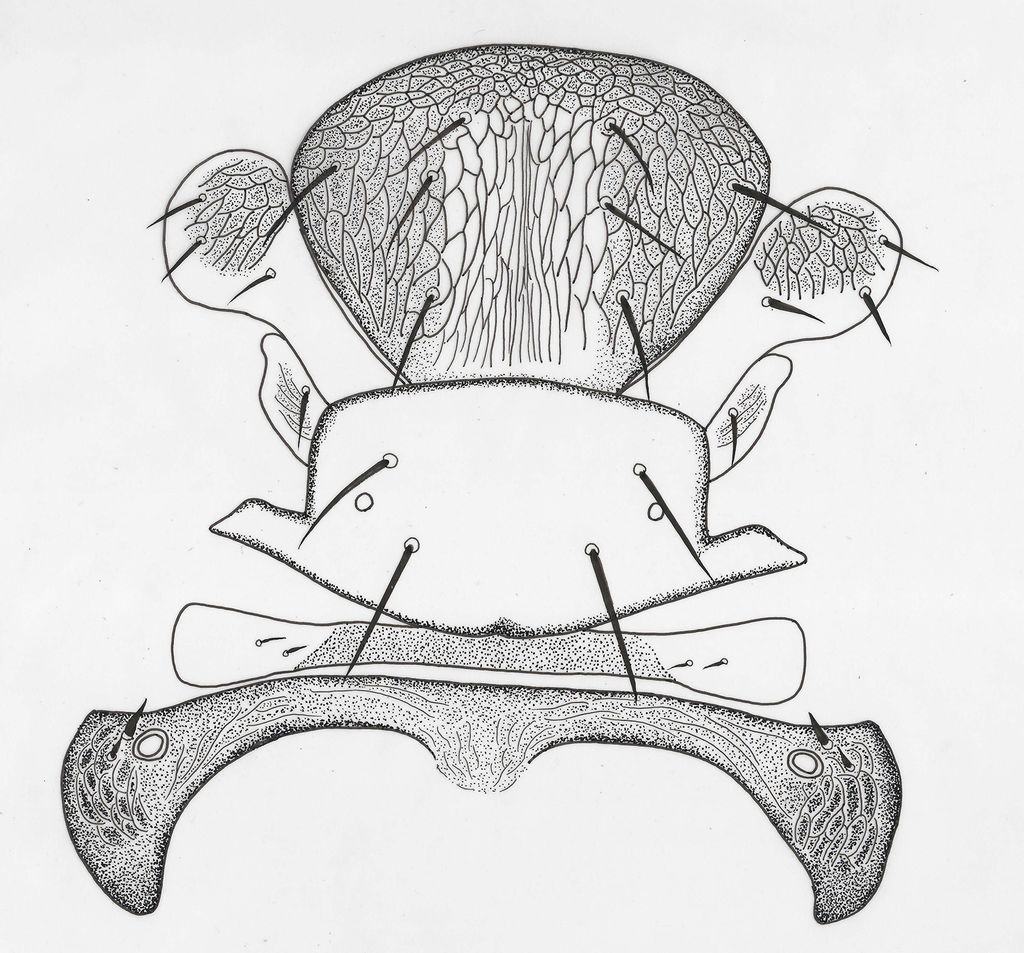
*Eretmocerus liangyihchoui* dorsal mesosoma, excluding pronotum

**Mesosoma** (Fig. 9​). Mid lobe of mesoscutum with 8 setae, anterior part with cellular reticulate sculpture, remainder with faint elongate reticulations; side lobe with 3 setae, anterior margins with faint reticulations; axilla with 1 long seta, weakly reticulate; scutellum with 4 setae, anterior pair length almost the same as posterior pair; 2 placoid sensilla placed close to the posterior pair of setae; scutellar reticulation weak; frenal arms normal, exceeding metanotum slightly.

**Wings** (Fig. 11). Fore wing 2.5-2.6× as long as maximum width of disc; longest posterior marginal fringe seta 0.2× disc width; base of wing with one seta, distal portion of costal cell with 4 setae; marginal vein short, 0.65× Submarginal vein length, with 3-4 larger setae; parastigma with 1-2 long setae; a group of setae forming 3-4 lines between marginal vein and linea calva; linea calva incomplete, closed basally by 10-13 setae; submarginal vein with 3 long setae, 1.53× as long as marginal vein and 2.7× stigmal vein; marginal vein 1.77× stigmal vein.

**Legs**. Mid tibial spur 0.5× basitarsus length. Hind tibial spur 0.3× basitarsus length.

**Figure 10.**
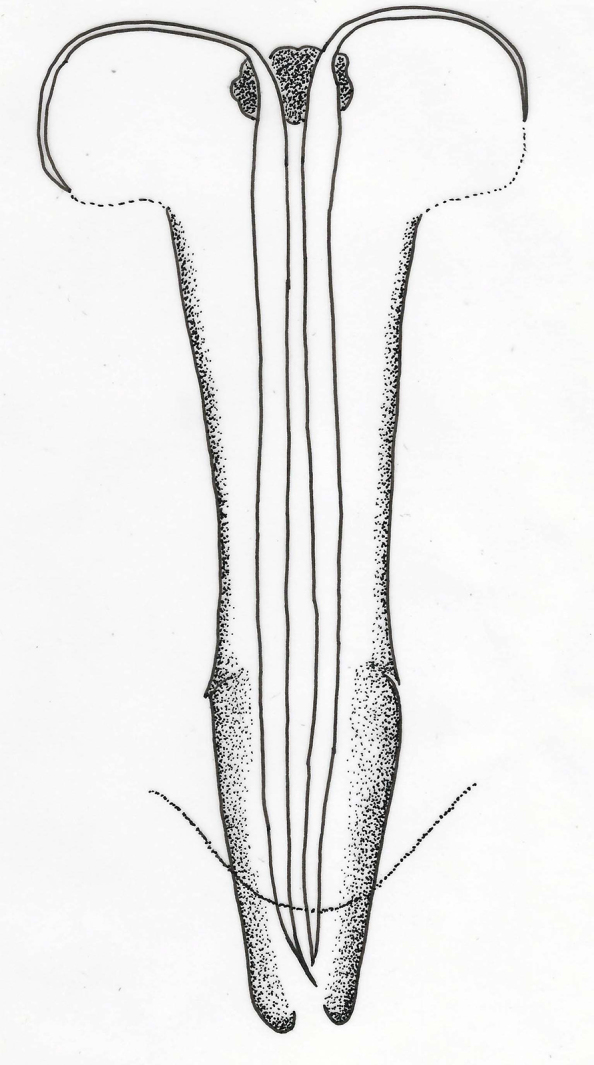
*Eretmocerus liangyihchoui* ovipositor

**Figure 11.**
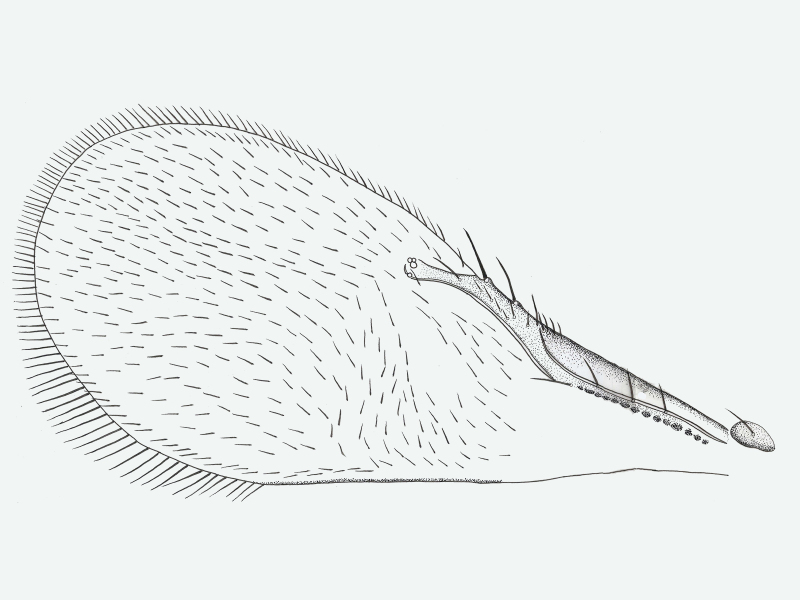
*Eretmocerus liangyihchoui* fore wing

**Gaster.** Gastral tergite 1 with reticulations on lateral margins; tergites 1–6 with paired setae as follows: 1, 2, 2, 2, 2, 1; syntergum (T7) with 4 setae; ovipositor (Fig. 10) normal, not exserted, 1.3× clava, longer than mid tibia; 2.0× scape.

**Male.** Unknown.

#### Diagnosis

*Eretmocerus liangyihchoui* **sp.n.** can be distinguished from other species in the genus by the following combination of characters: Mesoscutum with 8 setae; scape more than 0.66x clava length; propodeum dark brown.

#### Etymology

The species name *liangyihchoui* commemorates the late Dr Chou Liang-yih’s contribution to Aphelinidae studies in Taiwan.

#### Distribution

**TAIWAN**: Taipei City: Kungkuan, Mucha, Yangmingshan; New Taipei City: Ankeng; Taoyuan City: Linkou; Nantou County; Taichung City: Wufeng, TARI; Yunlin County: Huwei; Chiai City; Kaohsiung City: Fengshan.

#### Biology

**Host.**

Hemiptera: Aleyrodidae: *Aleurolobus rhododendri* Takahashi.

## Identification Keys

### Key to females of *Eretmocerus* species from Taiwan

**Table:** 

1	F1 of antenna anelliform (ring like)	2
–	F1 of antenna not anelliform; triangular, trapezoid, or longer than wide	3
2	Marginal vein longer than stigmal vein	*E. garrywardi* Ward **sp. n.**
–	Marginal vein length equal to stigmal vein length	*E. orientalis* Silvestri
3	Mesoscutum with 6 or more setae	4
–	Mesoscutum with fewer than 6 setae	10
4	Mesoscutum with 8 setae	*E. liangyihchoui* Shih **sp. n**
–	Mesoscutum with 6 setae	5
5	Propodeum brown	6
–	Propodeum yellow	7
6	Marginal fringe more than 0.25x maximum wing width	*E. queenslandensis* Schmidt & Naumann
–	Marginal fringe less than 0.25x maximum wing width	*E. tongxiaoensis* Shih & Polaszek
7	T3 with one pair of setae	8
–	T3 with two pairs of setae	9
8	T4 with one pair of setae	*E. trialeurodis* Hayat
–	T4 with two pairs of setae	*E. lannae* Shih & Polaszek
9	Clava cylindrical	*E. rui* Zolnerowich & Rose
–	Clava spatulate	*E. flavus* Krishnan & David
10	Mesoscutum with 2 setae	*E. bisetae* Hayat
–	Mesoscutum with 4 setae	11
11	F1 triangular	*E. furuhashii* Rose & Zolnerowich
–	F1 trapezoid or quadrate	12
12	Scutellum pale	*E. mundus* Mercet
–	Scutellum brown	*E. melanoscutum* Zolnerowich & Rose, 1998

## Supplementary Material

XML Treatment for Eretmocerus garrywardi

XML Treatment for Eretmocerus liangyihchoui
